# Discordance in Couples Pregnancy Intentions and Breastfeeding Duration: Results from the National Survey of Family Growth 2011–2013

**DOI:** 10.1155/2018/8568341

**Published:** 2018-07-24

**Authors:** Jordyn T. Wallenborn, Gregory Chambers, Elizabeth P. Lowery, Saba W. Masho

**Affiliations:** Virginia Commonwealth University, School of Medicine, Division of Epidemiology, Department of Family Medicine and Population Health, 830 East Main Street, Suite 821, P.O. Box 980212, Richmond, VA 23298-0212, USA

## Abstract

**Background:**

Parental disagreement in pregnancy intention elevates the risk of adverse health events for mother and child. However, research surrounding parental pregnancy intention discrepancies and breastfeeding duration is limited. This study aims to examine the relationship between couple's discordant pregnancy intention and breastfeeding duration.

**Methods:**

Data from the 2011–2013 National Survey of Family Growth was analyzed. Parental pregnancy intention was categorized as “intended by both parents,” “unintended by both parents,” “father intended and mother unintended,” and “father unintended and mother intended.” Breastfeeding duration was categorized as “never breastfed,” “breastfed less than six months,” and “breastfed at least six months.” Multinomial logistic regression, odds ratios, and 95% confidence intervals were calculated.

**Results:**

Couples with a concordant unintended pregnancy were more likely to have a child who was never breastfed or breastfed less than six months compared to couples with a concordant intended pregnancy. Similarly, couples with a discordant pregnancy were more likely to have a child who was never breastfed or breastfed less than six months.

**Conclusions:**

Findings from this study show a relationship between couples' pregnancy intentions and subsequent breastfeeding behaviors. Healthcare professionals should be cognizant of parents' differing opinions surrounding pregnancy intention and the implications on breastfeeding outcomes.

## 1. Introduction

Breastfeeding is considered the optimum source of nutrition for infants. Research has correlated breastfeeding with lower rates of upper respiratory infections, otitis media, and necrotizing enterocolitis [1, 2]. Breastfeeding is also linked to lower rates of childhood obesity, asthma, and dental caries [3–6]. Similarly, breastfeeding has health benefits for mothers. Not only can breastfeeding improve sleep quality and feelings of maternal well-being [7], but also breastfeeding duration is associated with a decreased risk for ovarian and breast cancer, type 2 diabetes, and an earlier return to prepregnancy weight [8, 9].

Despite the numerous benefits associated with breastfeeding, a small proportion of women breastfeed for the recommended duration. The American Academy of Pediatrics recommends exclusive breastfeeding through 6 months of age, followed by continued breastfeeding while supplementing with other foods throughout the first year of life [10]. However, in 2013, only 22% of mothers exclusively breastfed for 6 months. Further, when combining breastfeeding with other forms of feeding, only 52% of infants were receiving breast milk at 6 months [11].

While the choice to initiate or continue breastfeeding is a complex decision, research has identified a variety of factors that influence breastfeeding outcomes. Some of these factors include the father's support for breastfeeding, the breadth of support from the woman's social network, and how quickly the mother returns to work [12–15]. This is exemplified by a statement released by the Centers for Disease Control and Prevention which reported a lack of support for breastfeeding mothers from employers and communities [11]. Another factor associated with breastfeeding outcomes is maternal and paternal pregnancy intention [16, 17]. Specifically, research has found that maternal pregnancy intention is an important determinant of breastfeeding cessation [16]. Mothers with an unwanted pregnancy are less likely to initiate or continue breastfeeding [18]. Similarly, a recent cross-sectional study showed that a pregnancy unintended by the father was associated with a shorter breastfeeding duration [17].

While current research has independently investigated maternal and paternal pregnancy intention and subsequent breastfeeding practices, discordance in couples' pregnancy intention has not been explored. Previous literature has demonstrated that a couples' discordant pregnancy intention can impact perinatal outcomes. For example, discordant pregnancy intention has been associated with higher odds of rapid repeat pregnancy, which can lead to preterm birth, low birth weight, and neonatal death [19]. In addition, discordant pregnancy intention has been linked to lower rates of breastfeeding initiation, delayed prenatal care, increased smoking in pregnancy, and higher rates of preterm birth [20, 21]. Moreover, studies have suggested that pregnancy intentions can influence maternal behaviors and health outcomes [22, 23].

In 2010, nearly half (45%) of all pregnancies in the United States (US) were unintended [24]. In light of recent estimates of unintended pregnancies, understanding the implications of a couples' pregnancy intention on breastfeeding outcomes could provide important insight for interventions aimed at increasing breastfeeding rates. However, to the authors' knowledge, no study has examined the association between couples' pregnancy intention and breastfeeding duration. Therefore, the current study aims to investigate the association between couples' discordant pregnancy intention and breastfeeding duration.

## 2. Materials and Methods

The current study utilized data from the 2011–2013 National Survey of Family Growth (NSFG), which collects information on family life, marriage, pregnancy, and overall men and women's health. The US Department of Health and Human Services uses this information to help plan public health programs and other health services [25]. The survey was specifically created to provide national estimates and is conducted through in-person interviews and self-administered questionnaires. The response rate for recent data is estimated at 3%. Additional information on NSFG can be found elsewhere [26].

Analysis for the current study was restricted to women's first birth to reduce confounding factors related to breastfeeding subsequent children [27]. Women were also excluded if they were never pregnant, received help to become pregnant, or had missing information on the main outcome or exposure. The final analysis was conducted on 2,231 women. This study was approved as exempt by the Virginia Commonwealth University Institutional Review Board.

Breastfeeding duration, the outcome of interest, was defined as “never breastfed,” “breastfed less than 6 months,” or “breastfed at least 6 months.” Breastfeeding was assessed using 3 self-report survey items, where mothers reported breastfeeding duration in weeks. If the mother reported breastfeeding 1-25 weeks, she was categorized as breastfeeding less than 6 months, while if the mother reported breastfeeding 26 weeks or more, she was categorized as breastfeeding at least 6 months, which is consistent with national breastfeeding recommendations [11].

Couple pregnancy intention for the first live birth, the main exposure, was categorized as “intended by both parents (father + mother +),” “unintended by both parents (father – mother –),” “father intended and mother unintended (father + mother –),” and “father unintended and mother intended (father – mother +).” Unintended pregnancy was defined as a pregnancy that occurred too soon (e.g., mistimed) or was unwanted. Intended pregnancy included one that was (1) at the right time, (2) overdue, or (3) indifferent. Categorization was based on questions regarding pregnancy intention prior to contraception and is consistent with previous literature [19, 28].

Couples where both parents reported the pregnancy as intended were used as the reference category because previous literature has shown that intended pregnancies have better breastfeeding outcomes [16, 17].

Potential confounding factors were selected based on literature and availability in NSFG [15, 17, 29]. Sociodemographic factors considered included maternal age (≤ 19 years; 20-24 years; 25-29 years; 30-34 years; 35+ years), paternal age (18-24 years; 25-49 years), maternal race/ethnicity (non-Hispanic White; non-Hispanic Black; Hispanic; Non-Hispanic Other), marital status (married; not married), poverty level (0-99 percent; 100-199 percent; 200-399 percent; 400-700 percent), highest maternal educational attainment (less than high school; high school; some college or more), and maternal prepregnancy body mass index (BMI) (normal weight (15–24.9 kg/m2), overweight (25.0–29.9 kg/m2) and obese (≥30.0 kg/m2). Other factors including whether the mother was born outside the US (yes; no) and current religious affiliation (no religion; catholic; protestant; other) were also considered.

All analyses were conducted using SAS 9.4 statistical software to account for the complex survey design of NSFG. Descriptive statistics including unweighted frequencies and weighted percentages were used to describe the study population by couple pregnancy intention. Bivariate analyses were used to determine factors associated with breastfeeding duration. Effect modification by marital status (*p *= <.0001), paternal age (*p *= <.0001), and race/ethnicity (*p *= 0.9886) was tested in accordance to previous literature [17, 30, 31]. However, because they were not included in the a priori hypothesis, stratified analysis was not considered. Because marital status and race/ethnicity were effect modifiers, they were not considered as potential confounders. Multinomial logistic regression models were used to generate crude and adjusted odds ratios and 95% confidence intervals (CI). An iterative process was used to determine factors to include in the final parsimonious model such that any potential confounding factor that changed the crude estimate by at least 10% was included in the final model [32].

## 3. Results

Overall, the majority of the mothers in the sample were White, Non-Hispanic (54.0%), and married (56.5%) and had at least some college education (56.1%). The majority of respondents (50.6%) reported a concordant intended pregnancy and 27.0% reported a concordant unintended pregnancy. Less than a quarter of respondents reported a discordant pregnancy intention (father – mother +, 13.5%; father + mother –, 8.9%) (not shown in Tables 1–3). Couple pregnancy intention was associated with maternal age, paternal age, race/ethnicity, poverty level, marital status, highest educational attainment of the mother, whether the mother was born outside the US, and religion (Table 1). The bivariate analysis showed that all potential confounding factors were significantly associated with breastfeeding duration (Table 2).

The unadjusted analysis showed that couples with a concordant unintended pregnancy (father – mother –) had greater odds of having a child who was never breastfed or breastfed less than six months. Similarly, couples where the father reported an intended pregnancy and the mother reported an unintended pregnancy (father + mother –) had greater odds of having a child who was never breastfed or breastfed less than six months. No association was found among couples where the father reported an unintended pregnancy and the mother reported an intended pregnancy (father – mother +) (Table 3).

After adjusting for poverty level, maternal age, race/ethnicity, and whether the mother was born outside the US, the odds of never breastfeeding and breastfeeding less than six months were 148% and 80% higher among couples with a concordant unintended pregnancy (father – mother –) compared to couples with a concordant intended pregnancy. Similarly, the odds of never breastfeeding and breastfeeding less than six months were 79% and 49% higher among couples with a discordant pregnancy intention (father + mother –) compared to couples with a concordant intended pregnancy. Among couples with a discordant pregnancy intention (father – mother +), the odds of having a child who was breastfed less than six months were 130% higher compared to couples with a concordant intended pregnancy (Table 3).

## 4. Discussion

The current study found a relationship between couples' pregnancy intention and breastfeeding initiation and duration. Specifically, there were increased odds of never breastfeeding among couples with a concordant unintended pregnancy and among discordant couples (father – mother +). In addition, both categories of discordant pregnancy intention (father – mother +; father + mother –) and couples with a concordant unintended pregnancy had increased odds of breastfeeding a shorter duration. Further, the magnitude of association is much higher when the pregnancy was unintended by the father but the mother intends the pregnancy.

While there are no studies that examine couple's pregnancy intention and breastfeeding duration, results are consistent with previous literature on maternal and paternal pregnancy intention and breastfeeding duration. Specifically, research has shown that fathers with a mistimed or unintended pregnancy were more likely to have a child who was never breastfeed or breastfed a shorter duration [17]. Similarly, previous literature has demonstrated that mothers who have an unintended pregnancy are less likely to breastfeed at least 8 weeks [16]. A 2002 study of NSFG also found that women with unintended or mistimed pregnancies were more likely to never breastfeed or cease breastfeeding before 16 weeks [18].

The current study also found that couples with a concordant unintended pregnancy were more likely to have a child who was never breastfed or breastfed a shorter duration. This finding is consistent with a prior study that found that parents with a concordant unintended pregnancy were more likely to have an infant who was never breastfed [20]. The results also expand upon previous literature that demonstrated independent associations with maternal and paternal unintended pregnancies and a shorter breastfeeding duration [16–18].

The difference in breastfeeding outcomes for couples with a discordant pregnancy intention or a concordant unintended pregnancy may be due to the mediating influence of paternal support. Previous literature has shown that a pregnancy intended by the father is positively associated with paternal involvement [33, 34], which is linked to higher prevalence of exclusive breastfeeding at six months [35]. A study using the Early Childhood Longitudinal Study-Birth Cohort found that fathers who did not want the pregnancy were less likely to exhibit paternal warmth or engage in infant nurturing behaviors in the immediate postpartum period [33]. Further, pregnancies that are unintended by the father may lead to a lack of perceived support. As shown by a prospective cohort study conducted in Perth, Australia, perceived social support is significantly associated with breastfeeding duration [36].

The result may also be explained through maternal self-efficacy, which can be predicted by paternal support [37]. Self-efficacy relates to a person's confidence or belief that they can successfully accomplish a goal [38]. This belief directly relates to motivation, accomplishing a certain behavior, and emotional well-being [39]. Research suggests that social support can positively influence self-efficacy [40]. Therefore, the lack of paternal support as a result of an unintended pregnancy may lead to reduced self-efficacy in the mother. Studies have demonstrated a relationship between self-efficacy and breastfeeding outcomes. A prospective study reported that mothers with high self-efficacy were more likely to breastfeed a longer duration [41]. Self-efficacy can also be linked to unplanned pregnancies. Specifically, an experimental investigation found that women with low self-esteem had higher vulnerability to unplanned pregnancies [42].

To the authors' knowledge, this is the first study to examine the role of couples' pregnancy intention in breastfeeding duration. The current study used a nationally representative sample; therefore, results are generalizable to the US population. Lastly, the definition used for breastfeeding duration is consistent with the national recommendation to breastfeed for six months, which enables direct comparisons with other studies. Despite its strengths, this study is subject to certain limitations. First, the NSFG is a cross-sectional survey; therefore, causality cannot be inferred. Second, pregnancy intention and breastfeeding could be subject to social desirability and recall bias, which could lead to nondifferential misclassification and bias results towards the null. However, research has shown that self-report of breastfeeding duration is a valid and reliable measure [43]. Third, this study cannot distinguish exclusive breastfeeding from any breastfeeding; therefore, the definition of breastfeeding duration is not measuring full compliance with national breastfeeding recommendations. Lastly, potential confounding factors that could affect estimates including self-efficacy, perceived paternal support, school attendance, living arrangement, and lifestyle factors such as substance abuse could not be assessed due their unavailability in NSFG.

## 5. Conclusions

This study demonstrates an association between couples' pregnancy intention and breastfeeding duration. Due to the significant number of unplanned pregnancies in the US, the current findings could assist physicians, family planning advocates, and public health agencies in developing breastfeeding support programs. Developing stronger social support for breastfeeding, including outreach directed specifically to men, could assist in more infants reaching the feeding milestones set by Healthy People 2020. Longer maternity leave, breastfeeding facilities in the workplace, and support from healthcare workers may help women prolong breastfeeding. Future research should be directed at examining which factors can modify the relationship between couples' pregnancy intention and breastfeeding to provide points of action for future policy and programs. Lastly, research should be conducted among mothers with adolescent partners (<18 years old), as current literature is limited among this population.

## Figures and Tables

**Table 1:** Weighted distribution of characteristics by couple pregnancy intention among US mothers.

	**Total**	**Father +**	**Father –**	**Father +**	**Father –**	***P*-Value**
	**N=2231**	**Mother +**	**Mother –**	**Mother –**	**Mother +**	**(Chi-**
		**n=989**	**n=695**	**n=348**	**n=199**	**Square)**
**Maternal Age**						**<.0001**
≤ 19	1.3	8.8	74.9	10.4	5.8	
20-24	11.9	29.3	46.9	17.8	6.1	
25-29	19.3	37.6	36.6	17.8	8.0	
30-34	28.3	49.0	28.4	13.0	9.6	
35+	39.2	66.1	13.6	10.6	9.7	
**Paternal Age**						**<.0001**
18-24	42.9	31.4	42.9	18.4	7.3	
25-49	57.1	67.7	12.1	10.0	10.2	
**Race/Ethnicity**						**<.0001**
White, Non- Hispanic	14.1	31.4	38.9	22.9	6.8	
Black, Non- Hispanic	54.0	56.7	25.1	8.3	9.9	
Hispanic/Other	31.9	48.8	24.9	18.2	8.1	
**BMI**						0.2599
normal weight (15–24.9 kg/m2)	37.0	52.3	25.0	15.4	7.2	
overweight (25.0–29.9 kg/m2)	29.6	52.7	24.4	13.2	9.7	
Obese (≥30.0 kg/m2)	33.4	46.1	31.2	12.6	10.1	
**Poverty Level**						**<.0001**
0-99	33.1	41.2	30.5	17.2	11.2	
100-199	23.4	42.3	34.3	14.1	9.2	
200-399	26.2	58.3	24.2	11.3	6.2	
400-700	17.2	68.3	14.0	9.0	8.1	
**Marital Status**						**<.0001**
Married	56.5	64.3	18.8	9.7	7.1	
Other	43.5	32.8	37.6	18.5	11.1	
**Education**						**<.0001**
Less than H.S.	20.0	44.4	30.0	17.0	8.5	
High School	23.9	41.1	33.5	14.0	11.3	
Some College	56.1	56.9	23.1	12.1	8.0	
**Born Outside US**						**0.0012**
Yes	20.0	61.6	14.8	15.5	8.2	
No	80.0	47.9	30.0	13.0	9.1	
**Insurance Status**						**<.0001**
Private	50.6	60.8	21.1	10.5	7.5	
State Sponsored Health Plan	21.1	32.7	39.7	16.8	10.8	
Other Government Health Care	5.1	43.4	36.0	16.8	3.8	
None	23.2	46.3	26.2	16.3	11.2	
**Breastfeeding Duration**						**<.0001**
Never Breastfed	31.8	37.7	36.7	16.1	9.5	
Breastfed < 6 months	36.0	49.3	26.9	13.0	10.8	
Breastfed ≥ 6 months	32.3	64.7	17.6	11.6	6.1	
**Religion**						**0.0132**
No religion	18.3	48.1	33.0	12.0	6.9	
Catholic	23.8	59.9	19.5	11.9	8.6	
Protestant	49.3	46.1	30.0	15.0	8.9	
Other	8.5	55.8	17.5	12.7	14.1	

BMI: body mass index; US: the United States; HS: high school.

**Table 2:** Factors associated with breastfeeding duration among US mothers: NSFG 2011-2013.

	**Odds Ratio (95**%** CI)**	
	**Never Breastfed**	**Breastfed < 6 months**
**Maternal Age**		
≤ 19	3.14 (0.38-26.20)	2.08 (0.24-17.98)
20-24	1.43 (0.92-2.23)	1.30 (0.64-2.63)
25-29	**1.74 (1.46-2.08)**	**1.68 (1.04-2.69)**
30-34	1.00	1.00
35+	0.71 (0.44-1.14)	0.90 (0.64-1.26)
**Paternal Age**		
18-24	**2.04 (1.43-2.92)**	1.26 (0.98-1.63)
25-49	1.00	1.00
**Race**		
White, Non- Hispanic	1.00	1.00
Black, Non-Hispanic	**0.34 (0.26-0.44)**	0.85 (0.53-1.36)
Hispanic/Other	**0.26 (0.19-0.35)**	**0.51 (0.32-0.82)**
**BMI**		
normal weight (15–24.9 kg/m2)	1.00	1.00
overweight (25.0–29.9 kg/m2)	**1.37 (1.05-1.78)**	**1.30 (1.08-1.56)**
obese (≥30.0 kg/m2)	**1.80 (1.16-2.78)**	1.24 (0.88-1.75)
**Poverty Level (**%**)**		
0-99	1.00	1.00
100-199	0.78 (0.61-1.00)	0.93 (0.63-1.36)
200-399	**0.58 (0.41-0.80)**	0.85 (0.59-1.22)
400-700	**0.30 (0.16-0.55)**	0.89 (0.44-1.78)
**Marital Status**		
Married	1.00	1.00
Not Married	**1.86 (1.46-2.37)**	1.08 (0.82-1.42)
**Education**		
Less than HS	**2.18 (1.11-4.28)**	0.95 (0.49-1.86)
High School	**3.09 (1.93-4.96)**	**1.69 (1.20-2.37)**
Some College	1.00	1.00
**Born Outside US**		
Yes	**0.32 (0.24-0.45)**	0.61 (0.37-1.01)
No	1.00	1.00
**Insurance Status**		
Private	1.00	1.00
State Sponsored Health Plan	**2.61 (1.67-4.07)**	1.08 (0.67-1.73)
Other Government Health Care	**1.53 (1.05-2.23)**	1.54 (0.78-3.02)
None	1.23 (0.66-2.30)	**0.74 (0.59-0.94)**
**Religion**		
No religion	1.03 (0.73-1.45)	0.97 (0.63-1.50)
Catholic	0.57 (0.28-1.14)	0.80 (0.46-1.38)
Protestant	1.00	1.00
Other	**0.30 (0.20-0.45)**	**0.36 (0.20-0.66)**

CI: confidence interval; BMI: body mass index; US: the United States; HS: high school.

**Table 3:** Association between couple pregnancy intention and breastfeeding duration: NSFG 2011-2013.

	**Unadjusted Model ** **COR (95% CI)**		**Parsimonious Model** ^**a**^ **AOR (95% CI)**	
	**Never Breastfed**	**Breastfed<6 months**	**Never Breastfed**	**Breastfed <6 months**
Father + Mother +	Reference		Reference	
Father – Mother –	**3.58 (2.19-5.84)**	**2.00 (1.22-3.28)**	**2.58 (2.05-3.25)**	**1.78 (1.24-2.54)**
Father + Mother –	**2.39 (1.72-3.31)**	**1.47 (1.13-1.92)**	**1.98 (1.37-2.87)**	**1.43 (1.07-1.91)**
Father – Mother +	2.67 (0.79-9.06)	2.33 (0.99-5.46)	2.20 (0.71-6.82)	**2.37 (1.08-5.17)**

COR: crude odds ratio; AOR: adjusted odds ratio; CI: confidence interval.

Note: breastfeeding at least 6 months is the reference category.

^a^Parsimonious model controlling for current insurance, poverty level, maternal age, and if the mother was born outside the United States.

## Data Availability

Data is publicly available to everyone.
